# Sex-related DNA methylation is associated with inflammation and gene expression in the lungs of healthy individuals

**DOI:** 10.1038/s41598-024-65027-y

**Published:** 2024-06-20

**Authors:** Devki Patel, Joseph P. McElroy, Daniel Y. Weng, Kamel Sahar, Sarah A. Reisinger, Jo L. Freudenheim, Mark D. Wewers, Peter G. Shields, Min-Ae Song

**Affiliations:** 1 Division of Environmental Health Sciences, College of Public Health, The Ohio State University, Columbus, OH USA; 2 Comprehensive Cancer Center, The Ohio State University and James Cancer Hospital, Columbus, OH USA; 3 Department of Epidemiology and Environmental Health, School of Public Health and Health Professions, University at Buffalo, Buffalo, NY USA; 4 Pulmonary and Critical Care Medicine, Department of Internal Medicine, The Ohio State University, Columbus, OH USA; 5 Comprehensive Cancer Center, The Ohio State University Wexner Medical Center, Columbus, OH USA

**Keywords:** Genome-wide DNA methylation, Sex, Lung, Inflammation, Expression, Biomarkers, Risk factors, Epigenetics, DNA methylation

## Abstract

Lung cancer exhibits sex-biased molecular characteristics and epidemiological trends, suggesting a need for sex-specific approaches to understanding its etiology and treatment. DNA methylation alterations play critical roles in lung carcinogenesis and may serve as valuable biomarkers for precision medicine strategies. We employed the Infinium MethylationEPIC array to identify autosomal sex-related differentially methylated CpG sites (DM-CpGs) in lung epithelium of healthy individuals (32 females and 37 males) while controlling for age, BMI, and tobacco use. We correlated DM-CpGs with gene expression in lung epithelium and immune responses in bronchoalveolar lavage. We validated these DM-CpGs in lung tumors and adjacent normal tissue from The Cancer Genome Atlas (TCGA). Among 522 identified DM-CpGs, 61% were hypermethylated in females, predominantly located in promoter regions. These DM genes were implicated in cell-to-cell signaling, cellular function, transport, and lipid metabolism. Correlation analysis revealed sex-specific patterns between DM-CpGs and gene expression. Additionally, several DM-CpGs were correlated significantly with cytokines (IL-1β, IL-4, IL-12p70, and IFN-γ), macrophage, and lymphocyte counts. Also, some DM-CpGs were observed in TCGA lung adenocarcinoma, squamous cell carcinoma, and adjacent normal tissues. Our findings highlight sex-specific DNA methylation patterns in healthy lung epithelium and their associations with lung gene expression and lung immune biomarkers. These findings underscore the potential role of lung sex-related CpGs as epigenetic predispositions influencing sex disparities in lung cancer risk and outcomes, warranting further investigation for personalized lung cancer management strategies.

## Introduction

Sex-based differences in lung health, respiratory diseases, and lung cancer are increasingly recognized, suggesting a complex interplay of biological factors influencing disease susceptibility, progression, and treatment response^1–4^. While disparities in smoking rates contribute partially to these differences^5–8^, independent of smoking, there may be a sex-related susceptibility for lung cancer development^9,10^.

Altered DNA methylation signatures have been indicated as biomarkers for diagnosis and prognosis in lung cancer^11–14^. This is because altered DNA methylation is involved in several key biological regulatory pathways such as apoptosis, proliferation, cell cycle, DNA repair, tumor invasion, and metastasis^15^. Thus, sex-specific DNA methylation patterns may elucidate distinct molecular pathways underlying lung cancer development in males and females.

Despite the importance of sex-related DNA methylation in lung disease, genome-wide studies often overlook sex-specific methylation patterns by often controlling for sex in analysis, potentially missing critical insights into sex-related disparities^16^. While blood-based studies have shown sex-related differential methylation^16–20^, the lung-specific methylation landscape remains poorly understood. Since DNA methylation is highly tissue-type specific^21,22^ and regulates tissue-specific gene expression^13^, investigating sex-related methylation patterns in lung tissue is crucial for understanding sex-based differences in lung health.^8^.

Moreover, exploring sex-related DNA methylation in healthy individuals can uncover epigenetic biomarkers predisposing individuals to lung cancer, shedding light on potential targets for personalized treatments^23^. Despite higher lung cancer incidence and mortality rates in males, disparities in treatment response, particularly in immunotherapy, persist^24–26^. Sex-specific immune processes and mutation burdens partially explain these differences^24,27,28^. However, the contribution of DNA methylation to sex disparities in lung cancer remains largely unexplored. It is postulated that sex-related DNA methylation in lungs explains underlying molecular mechanisms of sex-related dimorphism in lung cancer that can be further utilized as new epigenetic targets unique to males or females. Moreover, the reversible nature of DNA methylation can serve as a guide to monitor cancer treatment response and further identify methylation patterns aimed to help reduce tumor load.

Here, we aim to study sex-related autosomal CpG methylation patterns in the lungs of healthy young adults and further investigate their associations with other lung biomarkers, including inflammation and gene expression. We validated the sex-related CpGs in lung tissues from The Cancer Genome Atlas (TCGA) to find persistent CpG methylation signatures from healthy to a disease state.

## Materials and methods

### Participants and recruitment

All methods were performed in accordance with relevant guidelines and regulations that were approved by the institutional review board at The Ohio State University. Young adults aged 20 to 30 were recruited from 2015 to 2019 in the central Ohio (Supplementary Table 1)^29–33^. All participants provided informed consent after receiving study information and an explanation, before undergoing bronchoscopy, bronchial brushing for DNA methylation and gene expression, and bronchoalveolar lavage (BAL) for immune cells and cytokines. Specifically, during the bronchoscopy procedure, sterile salt water was placed into the lung and immediately suctioned back, washing off immune cells and materials lining the area. Fluid was injected approximately 5–7 times, recovering samples at each lavage. Following the washing, a cytology brush was inserted to obtain brushes of grossly normal airway epithelium from the main bronchus of the lung.

### Cell counts and cytokines in bronchoalveolar lavage

BAL was centrifuged at 300*g* at 4 °C. The cell pellets and supernatant were separated for immune cell counts and cytokines, respectively. Cell counts were performed using Countess Automated Cell Counter (Invitrogen, Waltham, MA), and differential cell count was determined using Diff-Quik stained cytospins and light microscopy through a clinical histopathologist. The supernatant was analyzed for cytokines using Meso Scale Discovery^29,30^.

### Genome-wide DNA methylation and whole transcriptome array in lung epithelium

Allprep DNA/RNA kit (Qiagen, Germantown, MD) was used to extract total DNA from the bronchial brushings for the Infinium MethylationEPIC BeadChip (Illumina, San Diego, CA) and RNA for the GeneChip Human Transcriptome Array 2.0 (HTA 2.0; Affymetrix Inc., Santa Clara, CA) as we previously reported^29^. Briefly, raw data from the arrays were processed using Partek Genomics Suite 6.6 (Partek, St Louis, Missouri), and potential batch effects were removed using ANOVA (Analysis of Variance). For methylation data, raw data (idat files) were normalized by Subset-quantile Within Array Normalization (SWAN)^34,35^, and β-values were converted to M-values to control for heteroscedasticity by logit-transformation^36,37^. GRCh37/hg19 (Human Genome version 19) was used as a reference genome. Prior to statistical analysis, we excluded probes that were in the X or Y chromosomes, SNP-associated, off-target or had a detection *P* > 0.05^38,39^. For transcriptome data, raw data (CEL files) were normalized using Robust Multi-Array analysis (RMA)^40,41^ and log_2_ transformed in Partek. Analysis of covariance (ANOVA) was used to remove potential batch effects.

### Statistical analysis

All statistical analysis was performed through JMP Pro Version 15 (SAS, Cary, NC) and Partek. To identify differentially methylated CpGs, (DM-CpGs), a multivariate linear regression model was used by controlling for age, body mass index (BMI), and smoking status at Benjamini–Hochberg False Discovery Rate (FDR) < 0.1. To characterize the patterns of DM-CpGs, signatures were characterized by functional roles according to the following genomic locations: promoter, within 1500 bps of a transcription start site (TSS) (TSS1500), within 200 bps of a TSS (TSS200), 5ʹ untranslated regions (5ʹUTR), first exon (1stExon), body (non-promoter), 3ʹUTR (non-promoter); and intergenic regions^21,29^. The location of CpGs relative to the CpG island and surrounding regions were defined according to the Illumina annotation file. Further, downstream analyses were performed to correlate methylation levels of the DM-CpGs with inflammatory markers (cytokines and cells) and gene expression (CpG site within ± 1.5 Kb from transcript TSS using a Spearman correlation within each sex group. In this study, unique genes are referred to as not repeated or duplicated in the findings to avoid double counting results. For the downstream analyses of correlations between identified sex-related DM-CpGs and other lung biomarkers, a statistical significance was defined at FDR < 0.2.

### Comparison of DM-CpGs with The Cancer Genome Atlas (TCGA)

We utilized TCGA’s Lung Adenocarcinoma (LUAD, n = 29 for adjacent normal tissue and n = 433 for tumor tissues) and Lung Squamous Cell Carcinoma (LUSC, n = 42 for adjacent normal and n = 358 for tumor tissues (Supplementary Table 2). Of 522 DM-CpGs identified from this study of healthy individuals, we used a targeted approach and matched 275 CpGs that were available on the Illumina 450 K array for TCGA data to our list of sex-related DM-CpGs. To determine the DM-CpGs between females and males, we used one-way ANOVA. A statistically significant correlation for downstream analysis was defined at FDR < 0.2.

### Ingenuity pathway analysis

The DM-CpGs genes were classified by ingenuity pathway analysis (IPA; Ingenuity Systems, Qiagen) to investigate the potential biological roles of the signatures. The score [score =  − log_10_(*P* value)] was used to measure the probability of finding identified genes in a set of biological functions stored in the IPA Knowledge Base (IPKB) by chance alone.

### Ethics approval and consent to participate

The study was approved by the institutional review board at The Ohio State University, and all participants signed informed consent.

## Results

### Identification and characterization of DM-CpGs

**Figure 1:**
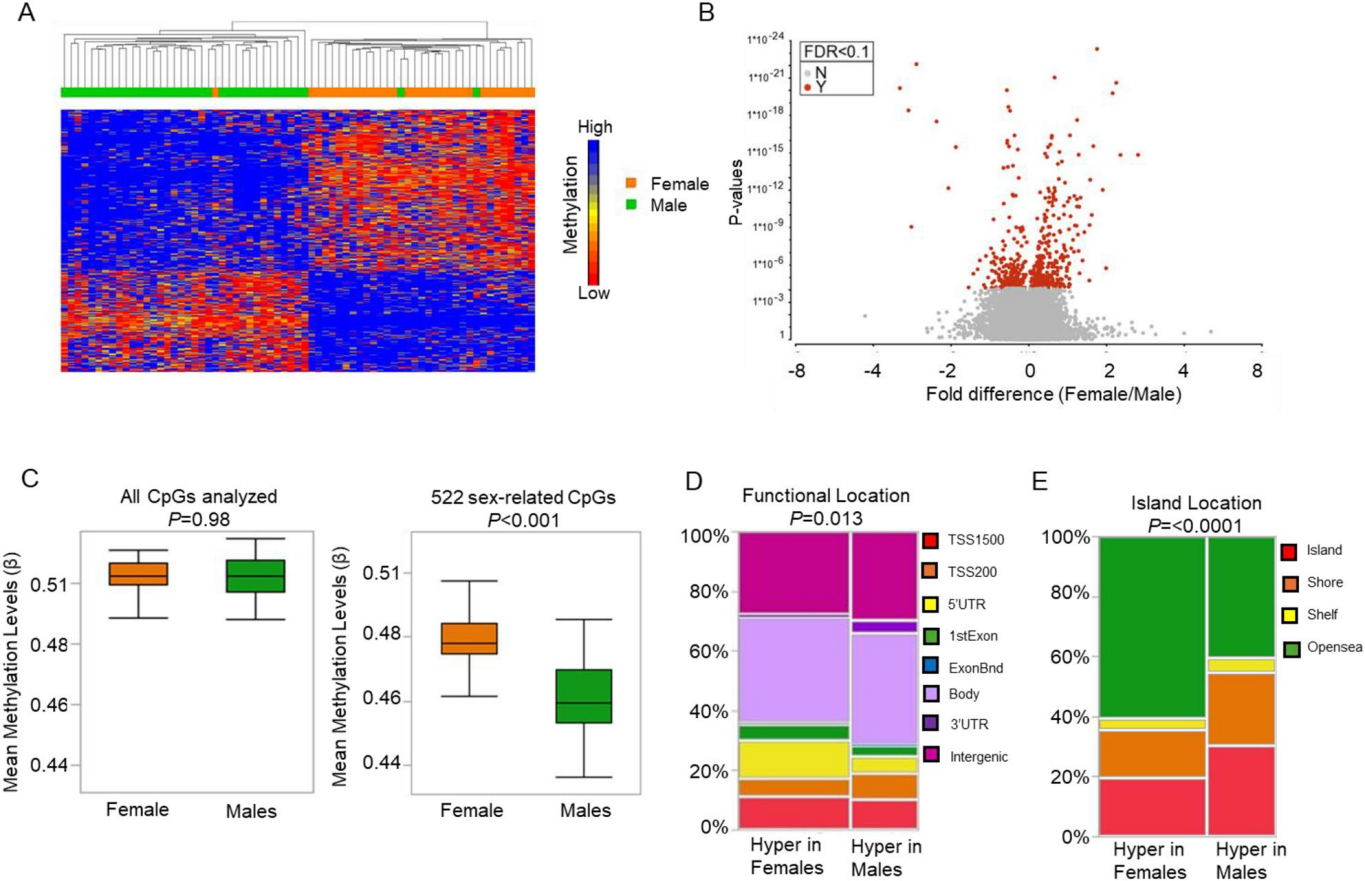
Differentially methylated (DM) CpGs between males and females. (**A**) Heatmap of β-values for 522 DM CpGs at FDR < 0.1. Green and orange blocks on the top of the maps represent males (n = 37) and females (n = 32), respectively. (**B**) Volcano plot, depicting fold change (females/males using β-values) vs. P-value. Red dots indicate 522 DM CpGs at FDR < 0.1. (**C**) Box plots indicating the mean β-values between males (green) and females (orange) using the entire CpGs analyzed (global) on the left and 522 DM CpGs on the right. (**D**) Mosaic graphs that classify the percentage of hypermethylated CpGs (# of hypermethylated CpGs out of 522 DM CpGs) in males on the right and females on the left into functional locations (TSS1500, TSS200, 5ʹUTR, 1stExon, ExonBnd, Body, 3ʹUTR intergenic regions) and CpG Island location (island, shore [north and south], shelf [north and south], and opensea).

We identified 522 sex-related DM-CpGs after adjusting for age, BMI, and tobacco use at FDR < 0.1. The majority of the DM-CpGs were hypermethylated in females compared to males (61.5% vs. 38.5%), as shown in the hierarchical clustering (Fig. 1A) and volcano plot (Fig. 1B). When compared to the mean β-values (reflecting global methylation) of 522 DM-CpGs, females had a significantly higher mean methylation level compared to males (0.48 vs. 0.46, *P* < 0.001 Fig. 1C right panel), while no difference was found for the entire set of CpGs analyzed (0.51 vs. 0.51, *P* = 0.98 Fig. 1C left panel). There was a statistically significantly different distribution of DM-CpGs across functional genomic locations (*P* = 0.013) and islands (*P* < 0.0001). Specifically, females had more hypermethylation of DM-CpGs in promoter regions, especially within 5ʹUTR, compared to hypermethylated CpGs in males (13.0% vs 6.0%) (Fig. 1D, Supplementary Table 3). In relation to CpG islands and their surrounding regions, females had more hypermethylation in Opensea (60.7% vs. 40.3%), while males had more hypermethylation in islands (30.3% vs. 19.6%) and shores (24.4% vs. 15.9%) (Fig. 1E, Supplementary Table 3).

### Potential biological roles of DM-CpGs genes and sex-related different patterns of correlation with gene expression

**Figure 2:**
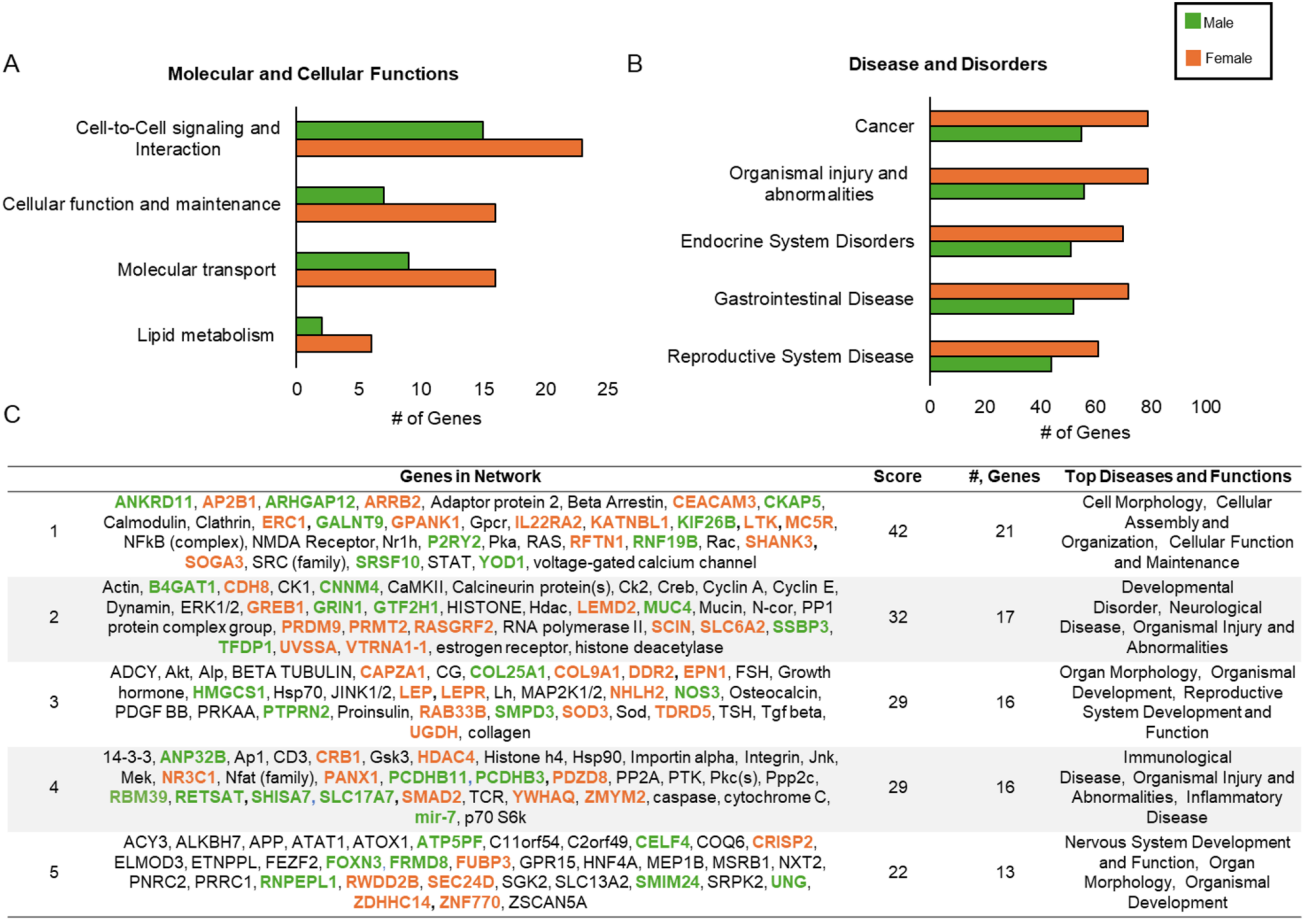
Classification of significant sex-related methylation genes by (**A**) molecular/cellular functions and (**B**) diseases/disorders, and (**C**) gene networks. (**A**,**B**) The green and orange bars indicate the number of hypermethylated sex-related genes in males and females. (**C**) The top five gene networks related to sex-related methylation genes. Genes highlighted in green are hypermethylated in males, while genes highlighted in orange are hypermethylated in females. Non-highlighted genes are not identified as sex-related but are involved in each network.

Sex-related methylation genes are involved in cell-to-cell signaling (n = 38), cellular function and maintenance (n = 23), molecular transport (n = 25), and lipid metabolism (n = 8) (Fig. 2A, Supplementary Table 4). The top diseases and disorders include cancer (n = 134), organismal injury and abnormalities (n = 135), endocrine system disorders (n = 121), gastrointestinal diseases (n = 124), and reproductive system disease (n = 105) (Fig. 2B, supplementary 4). The gene networks related to DM-CpGs genes are shown in Fig. 2C, and the top gene network is cell morphology, cellular assembly/organization, cellular function, and maintenance, including *ARRB2*, *NOS3*, *NR3C1*, and *P2RY2* that are known to play a role in respiratory system development and function.

**Figure 3:**
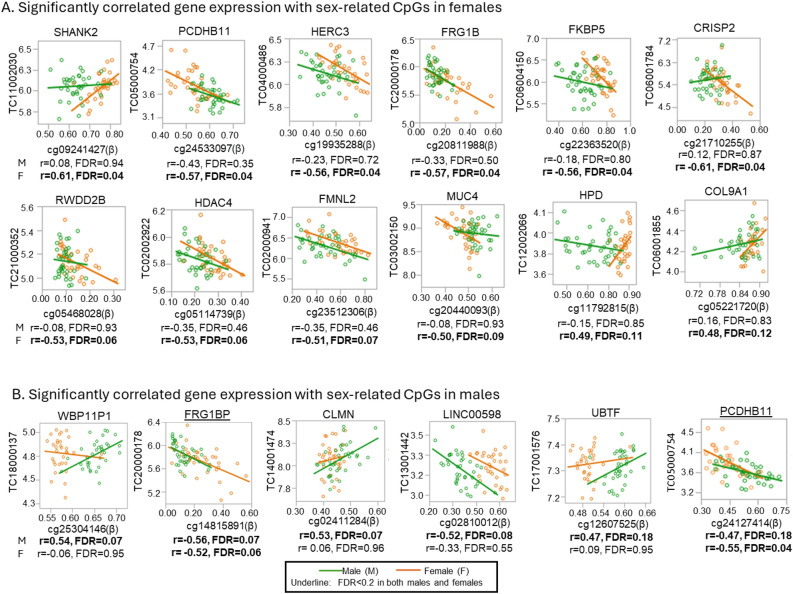
Significant correlation between sex-related CpGs and corresponding transcripts at FDR < 0.2 in females and males. Scatter plots show the correlation between sex-related CpGs (β-value) at the x-axis and transcript expression (log_2_) at the y-axis for hypermethylated CpGs in (**A**) females and (**B**) males. The green points and lines correspond to males and the orange points and lines correspond to females. Underlined genes are the ones that showed a significant correlation with a CpG in both males and females.

Of the identified 522 DM-CpGs, there were 412 with corresponding methylation and gene expression at transcription levels data. Females had twice as many unique genes significantly correlated with corresponding DM-CpGs than males. In a separate correlation analysis within males or females, females had 12 significant unique genes to be correlated with corresponding DM-CpGs, including cg09241427 with *SHANK2* (r = 0.61), cg24533097 with *PCDHB11* (r = − 0.57), cg19935288 with *HERC3* (r = − 0.56), cg20811988 with *FRG1B* (r = − 0.57), cg22363520 with *FKBP5* (r = − 0.56), cg21710255 with *CRISP2* (r = − 0.61), cg05468028 with *RWDD2B* (r = − 0.53), cg05114739 with *HDAC4* (r = − 0.53), cg23512306 with *FMNL2* (r = − 0.51), cg20440093 with *MUC4* (r = − 0.50), cg11792815 with *HPD* (r = 0.49), cg05221720 with *COL9A1* (r = 0.48) (Fig. 3, Table 1). In males, 6 unique genes were significantly correlated with corresponding DM− CpGs: cg25304146 with *WBP11P1* (r = 0.54), cg14815891 with *FRG1B* (r = − 0.56), cg02411284 with *CLMN* (r = 0.53), cg02810012 with *LINC00598* (r = − 0.52), cg12607525 with *UBTF* (r = 0.47), cg24127414 with *PCDHB11* (r = − 0.47) (Fig. 3B, Table 1). In both females and males, methylation levels of *FRG1B* and *PCDHB11* were significantly correlated with their corresponding gene expression (Fig. 3B).

**Table 1:** List of significantly correlated sex-related CpGs with corresponding transcripts at FDR < 0.2 in females and males.

CpG, IlluminaID	Functional location	CpG island	Genes	Transcript ID	r	*P*	FDR
Females							
cg09241427	5ʹUTR	Opeansea	SHANK2	TC11002030.hg.1	0.61	0.0002	0.04
cg24533097	TSS1500	N_Shore	PCDHB11	TC05000754.hg.1	− 0.57	0.0007	0.04
cg19935288	5ʹUTR	Opeansea	HERC3	TC04000486.hg.1	− 0.56	0.0008	0.04
cg20811988	Body	Island	FRG1BP	TC20000178.hg.1	− 0.57	0.0006	0.04
cg20811988	Body	Island	FRG1BP	TC20001182.hg.1	− 0.57	0.0007	0.04
cg22363520	Body	Opeansea	FKBP5	TC06004150.hg.1	− 0.56	0.0008	0.04
cg21710255	TSS1500	Opeansea	CRISP2	TC06001784.hg.1	− 0.61	0.0002	0.04
cg17731305	TSS1500	Opeansea	CRISP2	TC06001784.hg.1	− 0.58	0.0005	0.04
cg01706515	TSS1500	Opeansea	CRISP2	TC06001784.hg.1	− 0.57	0.0007	0.04
** cg24127414**	1stExon	N_Shore	PCDHB11	TC05000754.hg.1	− 0.55	0.001	0.04
** cg14815891**	Body	Island	FRG1BP	TC20001182.hg.1	− 0.54	0.0016	0.06
cg05468028	Body	Island	RWDD2B	TC21000352.hg.1	− 0.53	0.0019	0.06
cg05114739	Body	Opeansea	HDAC4	TC02002922.hg.1	− 0.53	0.0018	0.06
** cg14815891**	Body	Island	FRG1BP	TC20000178.hg.1	− 0.52	0.0021	0.06
cg23512306	Body	Opeansea	FMNL2	TC02000941.hg.1	− 0.51	0.0026	0.07
cg27188090	Body	Opeansea	FMNL2	TC02000941.hg.1	− 0.51	0.0031	0.08
cg20440093	Body	Opeansea	MUC4	TC03002150.hg.1	− 0.50	0.0037	0.09
cg11792815	Body	S_Shore	HPD	TC12002066.hg.1	0.49	0.0047	0.11
cg05221720	Body	N_Shore	COL9A1	TC06001855.hg.1	0.48	0.0056	0.12
Males							
cg25304146	Body	Opeansea	WBP11P1	TC18000137.hg.1	0.54	0.0005	0.07
** cg14815891**	Body	Island	FRG1BP	TC20000178.hg.1	− 0.56	0.0003	0.07
** cg14815891**	Body	Island	FRG1BP	TC20001182.hg.1	− 0.54	0.0006	0.07
cg02411284	Body	Opeansea	CLMN	TC14001474.hg.1	0.53	0.0007	0.07
cg02810012	Body	Opeansea	LINC00598	TC13001442.hg.1	− 0.52	0.001	0.08
cg12607525	Body	N_Shore	UBTF	TC17001576.hg.1	0.47	0.0032	0.18
** cg24127414**	1stExon	N_Shore	PCDHB11	TC05000754.hg.1	− 0.47	0.0035	0.18
cg02810012	Body	Opeansea	LINC00598	TC13001718.hg.1	− 0.47	0.0034	0.18

Bold CpGs show significant correlations with corresponding genes at FDR < 0.2 in both males and females.

### Different correlation patterns between DM-CpGs and inflammatory markers in bronchoalveolar lavage between males and females

**Figure 4:**
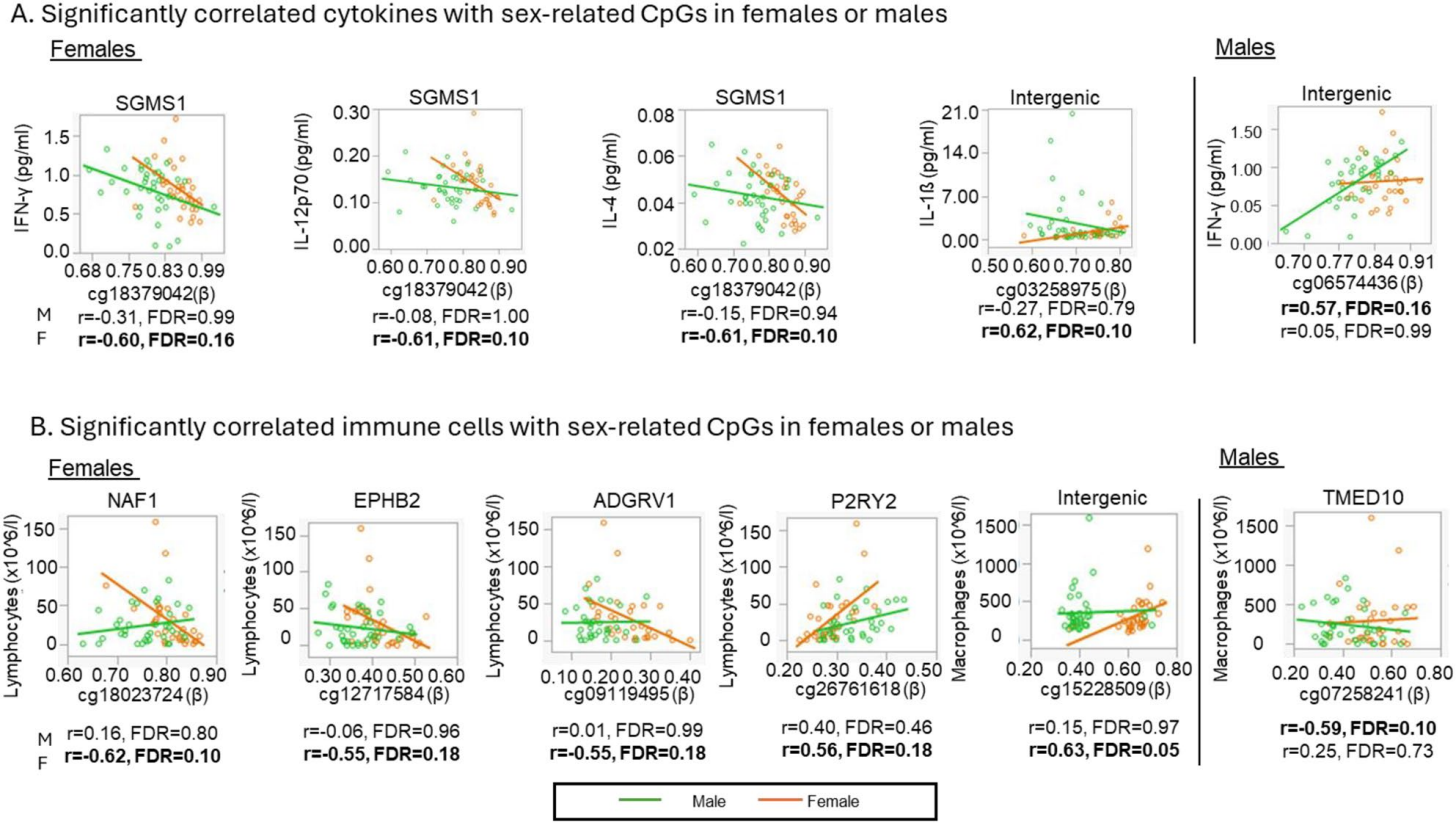
Significant sex-related CpGs with lung cytokines and cell count at FDR < 0.2 in females and males. Scatter plots show the correlation between hypermethylated sex-related CpGs at the x-axis and lung inflammatory responses, including (**A**) cytokines and (**B**) cell counts at the y-axis. The green and orange lines correspond to males and females, respectively.

In females only, one DM-CpG (cg18379042) in *SGMS1* was negatively correlated with IFN-γ (r = − 0.60), IL-12p70 (r = − 0.61), and IL-4 (r = − 0.61). One intergenic CpG (cg03258975) was positively correlated with IL-1ß (r = 0.62) (Fig. 4A, Table 2). Also, there were three DM-CpGs (cg18023724 in *NAF1*, cg12717584 in *EPHB2*, and cg09119495 in *ADGRV1*) negatively correlated with lymphocyte counts. In addition, there were two positively correlated DM-CpGs (cg15228509 within an intergenic region and cg26761618 in *P2RY2*) to macrophage and lymphocyte counts, respectively (Fig. 4B, Table 2). In a separate analysis of males, cg06574436 within an intergenic region was found to be positively correlated with IFN-γ (r = 0.57), while cg07258241 in *TMED10* were found to be negatively correlated with macrophages (r = − 0.59) (Fig. 4B, Table 2).

**Table 2:** List of significant sex-associated methylated CpG sites correlated with cytokines and immune cell count in bronchoalveolar lavage at FDR < 0.2

Genes	CpG island	Functional location	Illumina ID	Cytokines	r	*P*	FDR
Female							
Intergenic	N_Shore	–	cg03258975	IL-1ß	0.62	0.0002	0.10
SGMS1	Opeansea	5ʹUTR	cg18379042	IL-4	− 0.61	0.0002	0.10
SGMS1	Opeansea	5ʹUTR	cg18379042	IL-12p70	− 0.61	0.0002	0.10
SGMS1	Opeansea	5ʹUTR	cg18379042	IFN-γ	− 0.60	0.0003	0.16
Male							
Intergenic	Opeansea	–	cg06574436	IFN-γ (pg/ml)	0.57	0.0003	0.16

Genes	CpG Islands	Functional location	Illumina ID	Immune cells	r	*P*	FDR
Female							
Intergenic	Opeansea	–	cg15228509	macrophages	0.63	0.0001	0.05
NAF1	S_Shore	TSS1500	cg18023724	lymphocytes	− 0.62	0.0002	0.10
P2RY2	N_Shore	TSS1500	cg26761618	lymphocytes	0.56	0.0011	0.18
EPHB2	Opeansea	Body	cg12717584	lymphocytes	− 0.55	0.0014	0.18
ADGRV1	Opeansea	Body	cg09119495	lymphocytes	− 0.55	0.0013	0.18
Male							
TMED10	Opeansea	Body	cg07258241	macrophages	− 0.59	0.0002	0.10

### Replication of DM-CpGs in TCGA-LUAD and TCGA-LUSC datasets

**Figure 5:**
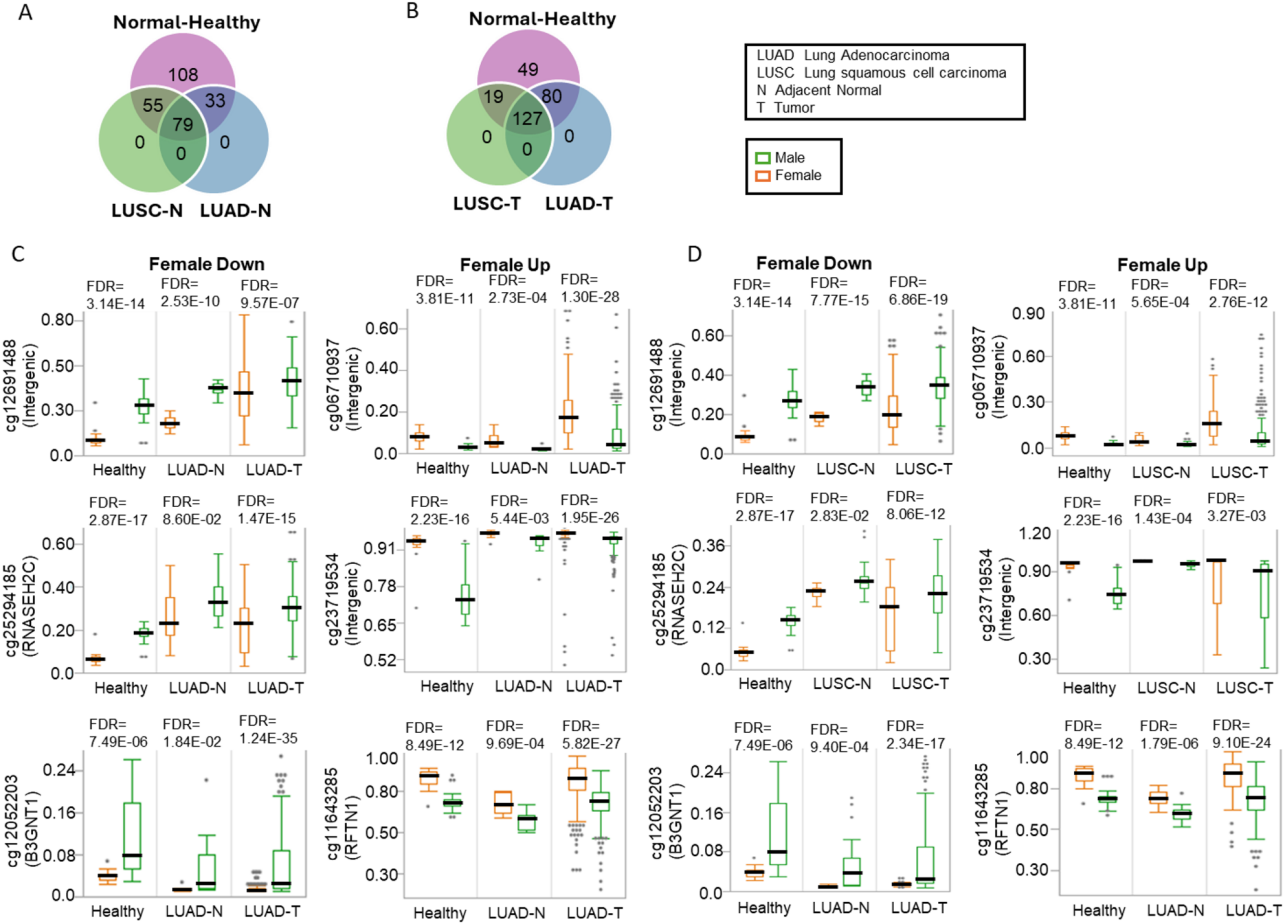
Validations of sex-related CpGs within TCGA datasets. Of 522 sex-related CpGs, 275 CpGs were available in the TCGA datasets. Venn diagrams showing the number of validated sex-related CpGs (with the same direction) in lung adenocarcinoma (LUAD) and lung squamous cell carcinoma (LUSC) from (**A**) adjacent normal tissues and (**B**) tumors, compared to the ones identified in this study (Healthy). (**C**) The top 3 sex-related CpGs with the greatest difference in methylation by direction. Those CpGs were compared to their corresponding tissue types within LUAD and LUSC data (tumor and normal-adjacent) (green for males and orange for females).

To replicate DM-CpGs identified in healthy individuals, we utilized TCGA-LUAD and TCGA-LUSC datasets for adjacent normal (n = 29 and n = 42) and tumor tissues (n = 433 and n = 358), respectively. Of the 522 DM-CpGs identified in the healthy individuals, 275 DM-CpGs were also found in the TCGA datasets. In normal adjacent tissue, 112 (with 95% in the expected direction) and 134 (with 96% in the expected direction) were found to be significantly differentially methylated between males and females LUAD and LUSC, respectively (Fig. 5A, Supplementary Table 5). In tumors, 207 DM-CpGs (with 95% in the expected direction) and 146 DM-CpGs (with 98% in the expected direction) were found to be validated in tissues from LUAD and LUSC, respectively (Fig. 5B on the right panel, Supplementary Table 5). There were 44 DM-CpGs that were uniquely identified in the lungs of healthy individuals, while there were 57 DM-CpGs to be validated in normal and tumor tissues from both LUAD and LUSC (Supplementary Fig. 1). Examples of validated DM-CpGs from LUAD and LUSC datasets are shown in Fig. 5C and D, respectively.

## Discussion

There is evidence of sex disparities in lung cancer regarding risk and treatment outcomes^1–4^. Sex-related biological differences for epigenetics in the lungs may explain this in part, but little is known about DNA methylation differences in healthy lungs before the carcinogenic process occurs. This cross-sectional study sheds light on sex-related DNA methylation differences in healthy lungs, potentially contributing to the understanding of sex disparities observed in lung cancer risk and treatment outcomes. The findings suggest that females exhibit increased gene methylation compared to males, with enrichment in pathways related to cancer and reproductive functions. These pathways, including cell-to-cell signaling, cellular function, transport, lipid metabolism, and cell morphology and maintenance, are crucial for disease progression, possibly implicating them in the observed sex disparities in lung cancer. Furthermore, the study establishes correlations between some DM-CpGs and gene expression in the lungs, providing evidence for the biological impact of sex-related DNA methylation. These results were validated by replicating the findings in adjacent non-tumor lung tissue and tumors of both males and females from the TCGA dataset, further supporting the relevance of these epigenetic differences in lung health and disease.

To our knowledge, this study is the first to identify sex-related DNA methylation in the lungs of healthy young adults, which was also observed in TCGA data. Some of the sex-related methylation genes, including *ARRB2*^24^, *NOS3*^41^, *NR3C1*^42^, and *P2RY2*^43^, are known to be involved in respiratory system development and function. Many of these findings are consistent with existing literature. For example, 6 separate studies showed sex-related DM-CpGs matched with 80 (98% concordance), 28 (100% concordance), 55 (100% concordance), 65 (100% concordance), 70 (100% concordance), and 145 (100% concordance) in various tissues from healthy individuals, such as in brain tissues^44^, in myoblasts/myotubes^31^ in pancreatic islets^45^, in leukocytes^20^ in whole blood^17^ in cord blood^35^ (Supplementary Table 6). Thus, some DM-CpGs may be used as robust sex-related methylation markers across tissues, but still, many lung DM-CpGs seem to be tissue-specific^37^.

In this study, we observed more hypermethylation patterns, especially within promoter regions, in females compared to males, consistent with previous findings^8,17,18,20,35,46,47^. This may indicate that female-biased hypermethylation may result in sex hormones-related gene regulation, given that sex hormones (i.e., estrogen) play an essential role in lung carcinogenesis and prognosis^48,49^. In this study, we found that there was sex-related differential methylation that correlated with gene expression for *SHANK2*^50^ and *HDAC4*^51^ which are known to be regulated by sex hormones. *SHANK2* is a scaffold gene that plays an oncogenic role in lung cancer^52^. *HDAC4* is a histone deacetylase that interacts with transcription factors, altering gene expression^53^. *HDAC4* promotes the progression of lung cancer by promoting epithelial–mesenchymal transition progress^54^. Also, sex-related methylation genes in this study included 105 reproductive system-related genes. Thus, our findings support further investigation of possible interactions between sex hormones and sex-related DNA methylation to address sex-based dimorphism in lung cancer.

Some of the DM-CpGs were correlated with lung immune cells and cytokines. A study of cancers showed a correlation between global DNA methylation and immune evasion in lung cancer, so that DNA methylation alterations may be pathologically related to immune evasion and may be a marker in precision immunotherapy^55^. Herein, we observed lung cytokines and immune cells to be correlated with some DM-CpG differently between males and females. For example, in females only, cg18379042 in *SGMS1* was significantly correlated with type 1 (IL-12p70 and IFN-γ) and type 2 (IL-4) cytokines. *SGMS1* is a key enzyme involved in the biosynthesis of sphingomyelins which plays a critical role in proliferation, apoptosis, membrane mobility, and airway smooth muscle functions^56^. Thus, DM-CpGs may explain sex-related disparities in lung cancer incidence and outcome by mediating different immune responses between males and females. As a follow-up analysis, we examined if there was an enrichment of cancer-related genes in our sex-related CpG genes by comparing our list of significant genes for over-representation of “lung cancer genes” from Ingenuity Pathway Analysis (IPA) (32.8% vs. 26.1%, Fisher’s Exact test *P* = 0.012) and “cancer genes” from the Catalogue of Somatic Mutations In Cancer (COSMIC) database (5.2% vs. 2.7%, Fisher’s Exact test *P* = 0.016), also indicating that sex-related methylation may play a role in cancer.

It is important to note that there are a few limitations to this study that should be considered in the interpretation of its findings. Small numbers precluded an analysis by tobacco group use (including no use), so DM-CpGs were identified after controlling for smoking status. Thus, we could not evaluate a possible important effect of lung toxicant exposure. Also, the sample size was too small to assess effects by BMI or age, and other possible intrinsic (i.e., mutations) and extrinsic factors, including environmental and occupational exposure. Moreover, the study only included young, healthy participants, which may limit the generalizability of the findings to broader populations. We utilized bronchial brushing, which is known to be mostly composed of epithelium cells, but we could not pathologically confirm the cellular composition of collected tissues. However, we confirmed that none of the estimated immune cell proportions, using Houseman et al.’s method (a gold standard for adjusting immune cell compositions in the blood), was associated with sex. Lastly, due to the cross-sectional design, causal relationships between differential methylation and other biomarkers could not be established. Despite these limitations, the study also had several strengths. It focused on the lungs as the target organ, providing insights into differential methylation patterns without morphological alterations or significant respiratory diseases. The comprehensive analysis of differential methylation and its biological implications, including inflammation and gene expression, adds depth to our understanding of epigenetic regulation in the lungs. Furthermore, the replication of some findings in independent datasets, including lung cancer datasets, enhances the robustness of the study’s conclusions.

In summary, the study contributes valuable insights into autosomal differential methylation in the lungs between sexes among young and healthy individuals. The correlations between some differential methylation sites and lung immune system biomarkers suggest potential implications for understanding sex-based dimorphism in lung cancer. However, further research addressing the study’s limitations and exploring causal relationships is warranted to fully elucidate the role of epigenetic differences in lung health and disease.

### Supplementary Information

Supplementary Table 1.

Supplementary Table 2.

Supplementary Table 3.

Supplementary Table 4.

Supplementary Table 5.

Supplementary Table 6.

Supplementary Figure 1.

## Data Availability

The datasets generated during and/or analyzed during the current study are available from the corresponding author upon reasonable request.
