# Supplementary material for: Sex-related DNA methylation is associated with inflammation and gene expression in the lungs of healthy individuals
Source: Sci Rep. 2024 Jun 20;14:14280. doi: 10.1038/s41598-024-65027-y (PMC11190195; doi:10.1038/s41598-024-65027-y)
Supplement: Supplementary file 7 — Supplementary Figure 1. [file 41598_2024_65027_MOESM7_ESM.pptx]

## Slide 1
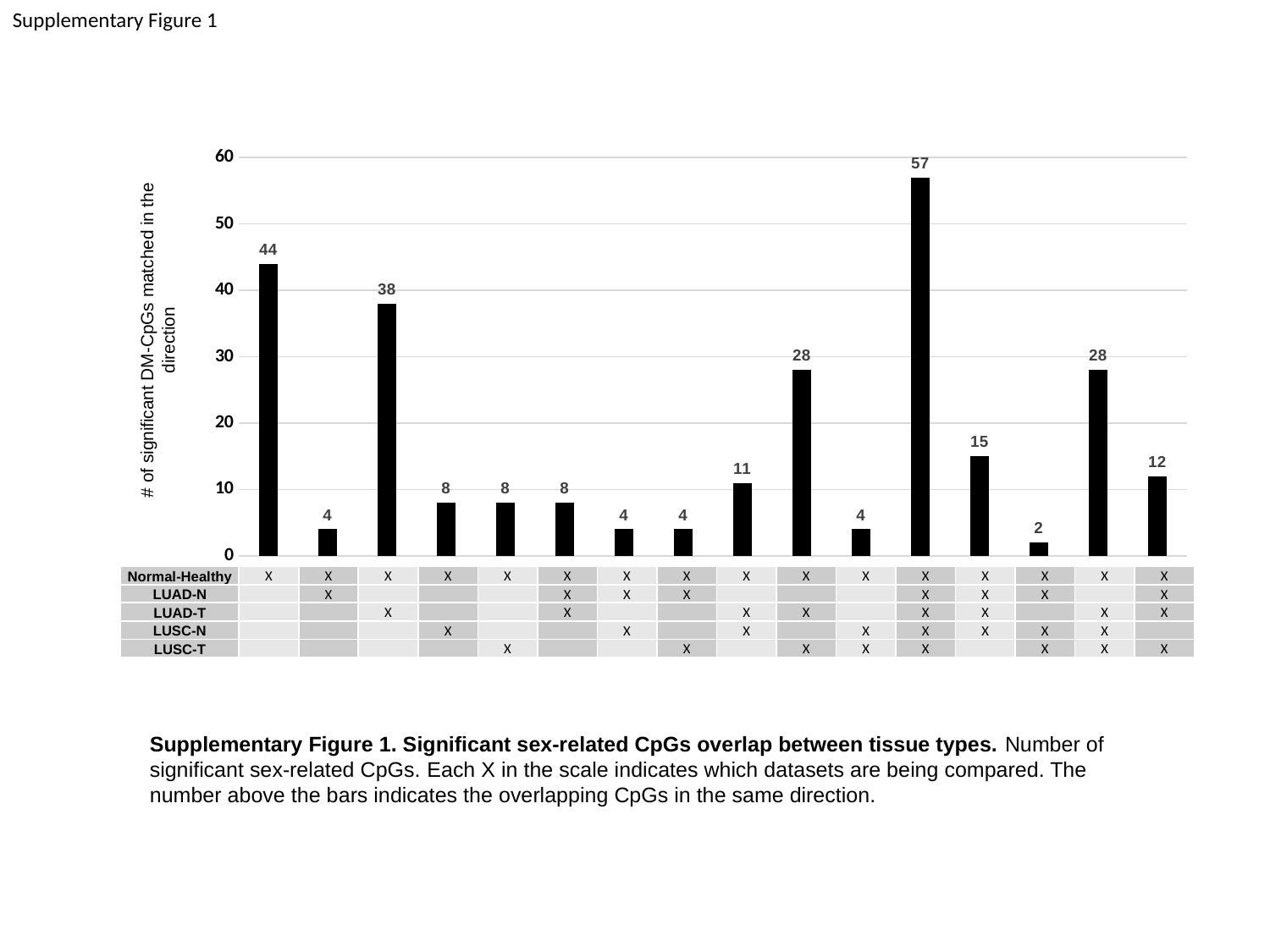

Supplementary Figure 1
### Chart
| Category | |
|---|---|
| Healthy | 44.0 |
| LUAD -N | 4.0 |
| LUAD-T | 38.0 |
| LUSC -N | 8.0 |
| LUSC-T | 8.0 |
| LUAD -N, LUAD-T | 8.0 |
| LUAD -N, LUSC -N | 4.0 |
| LUAD -N, LUSC-T | 4.0 |
| LUAD-T, LUSC -N | 11.0 |
| LUAD-T, LUSC -T | 28.0 |
| LUSC -N, LUSC-T | 4.0 |
| LUAD -N, LUAD-T, LUSC -N, LUSC-T | 57.0 |
| LUAD -N, LUAD-T, LUSC -N | 15.0 |
| LUAD -N, LUSC -N, LUSC-T | 2.0 |
| LUAD-T, LUSC -N, LUSC -T | 28.0 |
| LUAD -N, LUAD-T, LUSC-T | 12.0 |# of significant DM-CpGs matched in the direction
| Normal-Healthy | X | X | X | X | X | X | X | X | X | X | X | X | X | X | X | X |
| --- | --- | --- | --- | --- | --- | --- | --- | --- | --- | --- | --- | --- | --- | --- | --- | --- |
| LUAD-N | | X | | | | X | X | X | | | | X | X | X | | X |
| LUAD-T | | | X | | | X | | | X | X | | X | X | | X | X |
| LUSC-N | | | | X | | | X | | X | | X | X | X | X | X | |
| LUSC-T | | | | | X | | | X | | X | X | X | | X | X | X |
Supplementary Figure 1. Significant sex-related CpGs overlap between tissue types. Number of significant sex-related CpGs. Each X in the scale indicates which datasets are being compared. The number above the bars indicates the overlapping CpGs in the same direction.
